# Mica/Epoxy-Composites in the Electrical Industry: Applications, Composites for Insulation, and Investigations on Failure Mechanisms for Prospective Optimizations

**DOI:** 10.3390/polym8050201

**Published:** 2016-05-20

**Authors:** Natascha Andraschek, Andrea Johanna Wanner, Catharina Ebner, Gisbert Riess

**Affiliations:** 1 Polymer Competence Center Leoben (PCCL), Roseggerstraße 12, Leoben 8700, Austria; andreajohanna.wanner@pccl.at; 2 Chair of Polymer Chemistry, Montan University Leoben, Otto-Glöckl-Strasse 2, Leoben 8700, Austria; catharina.ebner@unileoben.ac.at (C.E.); gisbert.riess@unileoben.ac.at (G.R.)

**Keywords:** mica, mica/epoxy-composites, insulation materials, failure mechanisms, electrical insulation

## Abstract

The investigation of mica and mica/epoxy-composites has always been of high importance and has gained increased attention in recent years due to their significant role as insulation material in the electrical industry. Electrical insulation represents a key factor regarding the reliability and lifespan of high voltage rotating machines. As the demand for generating power plants is increasing, rotating machines are of intrinsic importance to the electrical energy supply. Therefore, impeccable functioning is of immense importance for both the producers of high voltage machines as well as the energy suppliers. Thus, cost reduction caused by shorter maintenance times and higher operational lifespan has become the focus of attention. Besides the electrical properties, composites should offer compatible chemical and mechanical, as well as thermal characteristics for their usage in insulating systems. Furthermore, knowledge of several occurring stresses leading to the final breakdown of the whole insulation is required. This review aims to give an overview of the properties of pure components, the composite, and the possible occurring failure mechanisms which can lead to a full understanding of insulation materials for high voltage rotating machines and therefore establish a basis for prospective optimizations.

## 1. Definition, Nomenclature, Composition, and Occurrence

Mica is a general term for a mineralogical group with common structural and chemical properties. From the mineralogical point of view, the term mica is not distinctly defined. Generally, it is assigned to the group of phylites, but according to the literature, it is referred to as its own group of so called hydro silicates [1,2,3,4].

The principle of classification of micas and therefore the nomenclature is based on chemical composition and generalized crystal-structure determination. Physical properties are discarded, since they do not offer the opportunity to distinctly differentiate between the diverse mica types. Therefore, the classification is based on the chemical data [5].

Despite a huge number of varieties, micas build their own mineral family with similar characteristics, which is represented in the following formula (general formula of mica) [6]:

**XY_2_·(OH, F)_2_·Z**´**Z_3_**´´**O_10_** (1)

This applies: **X** = K^+^, Na^+^, Rb^+^ and Cs^+^ in some cases as well; Ba^2+^ with a coordination number 12, **Y** = Al^3+^, Fe^3+^, Cr^3+^, Mn^3+^, V^3+^, Ti^3+^, FE^2+^, Mn^2+^ and Li^+^; rarely Zn^2+^ with a co-ordination number of 6, **Z**′= Al^3+^, often Si^4+^, as well as Fe^3+^, Mn^3+^ and Ti^3+^; with a co-ordination number 4 and **Z**′′ = only Si^4+^ with a co-ordination number 4.

The crystallo-chemical formula should be composed of chemical data, density, and cell data. The procedure of the formula calculation is recommended as follows, presuming that only chemical data is available: (1) If a solid determination of H_2_O is given, the formula should have regard to 12 O + F atoms; (2) In case no determination of H_2_O took place, as in electron-microprobes analyses, the assumption of an idealized ion group is necessary, which includes the fact that the formula is based on 22 positive charges; (3) Based on no determination of H_2_O and the presumption, that a later oxidation caused deprotonation of the anion group, the formula should consist of 22 + z positive charges, where z is defined as the quantity of trivalent iron [7,8,9].

Mica belongs to the monoclinic system. Formerly it was attributed to the hexagonal system due to its hexagonal crystal figurine. Accurate measurements however have shown that the angles are not exactly 120°. The hardness of mica is between 2.1 and 2.5 on the hardness scale which is based on the hardness of diamond with a value of 10 as a reference value; therefore it is harder than halite and gypsum, but softer than calcite.

Mica is widely spread in nature, but in hardly any provenance is it promoted in such an amount, respectively size, which is required for technical application. Among various amounts of mica types, only three types are of importance for electrical engineering, namely muscovite and phlogopite, and in small amounts biotite as well [10]. Muscovite, which is the most common and therefore the most important natural mica is represented in the following formula (muscovite) [6]:

**K·Al_2_·AlSi_3_·O_10_·(OH)_2_** (2)

The gaps in the formula expose the different parts which belong to each with regard to their ions. The most significant consideration from a chemical point of view is the fact that each 22 positive as well as negative charges occur. Within the scope of crystallo-chemical laws, the charges can be exchanged to build the different types of mica groups [6].

Muscovite is classified as a potassium–aluminum–double–silicate. The staining is reddish, as well as white or green in different shades, sometimes brown, partly clear, in some cases with reddish or black spots [10]. Phlogopite or so called chalk mica differs from muscovite by two Al^3+^ which have been exchanged for three Mg^2+^-ions and is represented by the formula (phlogopite) [6]:

**K·Mg_3_·AlSi_3_·O_10_·(OH)_2_** (3)

Phologite, which is also known as amber, is a chemically complex potassium-magnesium-aluminum-iron-double silicate and contains a slight amount of crystal- or constitution water compared to the previously mentioned muscovite. Hereby the crumbliness, which is characterized as softness, might be crucial and as well may be the reason for higher temperature stability. Compared to muscovite, which will calcinate between 600 and 650 °C, resulting in a loss of the solid crystal structure, phologite remains nearly undamaged in this temperature range. The calcination of the constitution water is the reason for blurring of the so far totally lucent crystal. The higher temperature stability (900 to 1000 °C) of phlogopite is the reason for its application in heaters. The color is amber to even reddish-amber [10].

Biotite or ferric mica (the ordinary black mica) is a phlogopite, which possesses iron ions in exchange for some of the magnesium (biotite) [6]:

**K·(Mg, Fe)_3_·AlSi_3_·O_10_·(OH)_2_** (4)

The chemical composition of biotite is very complicated and therefore does not really arouse the interest of electrical engineers. It occurs in huge crystals, its coloration is nearly always quite dark, mostly totally black. Due to the very dark staining, the occurring inclusions are hardly distinguishable, sometimes not at all. The application of biotite for electrochemical purposes is troublesome and met with failure due to its high surface conductivity within the layers. In addition the dielectric strength which is perpendicular to the crystal face is subjected to extraordinary fluctuations. The reasons for this are metallic, invisible inclusions and microscopic small cuts [10].

Besides the listed mica types there are further possibilities of substitution with relatively rare ions like Cr, V, Ta, Cs, and Rb [4,11].

## 2. Physical and Chemical Properties of Mica

The different physical and chemical properties of the commercially available mica types muscovite and phlogopite are summarized in Table 1.

These properties show that mica is an ideal material with a huge number of optimal properties covering many aspects. Especially for the purpose of electrical insulation mica exceeds all comparable materials due to its extremely high temperature resistance and low coefficient of thermal expansion.

The melting temperature of natural mica is around 1200–1300 °C. The high melting temperature, however, does not say anything about the temperature resistance. Since natural mica is a hydro silicate, it usually contains a certain amount of crystal-bound water. Depending on the chemical structure, calcination occurs at a particular temperature, which means, that the crystal bond breaks open, water becomes free, and some of the original properties undergo changes. Therefore, the calcination temperature is an important temperature for the quality evaluation of mica [6]. It is not flammable, has good dielectric properties and a very high dielectric strength. Furthermore, it is tracking resistant and corona stable and therefore the only choice when it comes to application in high voltage insulations. Last but not least, mica also meets the requirements concerning radiation resistance.

## 3. Mica in Technical Application

The biggest deposits of mica are found in India (muscovite), in Canada (phlogopite), in Argentina and Brasilia (muscovite), in Madagascar (phlogopite), in Africa (muscovite) and in the USA (muscovite) [6].

As previously mentioned, mica is a natural product which is commercially available in huge pieces as so-called block mica. By cleaving the block mica into thin layers the split mica occurs [12].

The better quality mica variety, mostly in the form of mica splittings, is mainly used in the electrical industry. In the seventies mica splittings were largely used for the manufacturing of vacuum tubes and condensers [13]. Splittings mainly serve the production of so called built-up mica, which is also known as micanite and “Mikafolium”. In this form it is used in the electrical industry and on the strength of its special properties it cannot be replaced by other materials [14,15,16].

The commercial value of mica depends on two main factors. The first is its size, which can be easily controlled by experienced producers. The second is its quality, whose diagnosis requires long term experts as its determination affords lots of experience. For the quality, three things are decisive: (1) the basic color; (2) the purity, and (3) the absence of errors in the crystal. The best mica should gleam in light-pink shades at a thickness of 0.1 mm, be free from inclusions, and be planar as well as free of cracks [10].

In addition, many experiments concerning the utilization of lower quality micas were performed [15,16,17,18,19].

Table 2 shows that the huge era of application of mica initially occurred due to the progress in the electrical engineering area.

In addition to this listing, the huge scope of application of several so-called “Mahlglimmer” as well as mica powder, used for attainment of particular effects, is the following (not only electro technical applications):

- Addition of colors (color industry)
- Filling material (rubber industry)
- Filling material (for molding compounds)
- Silky gloss (wallpaper production)
- Sheatings of welding rods
- Addition for exterior rendering (construction industry)

Mica powder exceeds other flake-shaped silica powders mainly because its flakes are quite thin, very elastic, smooth, and acid resistant. Furthermore it displays a high tear resistance [20,21].

## 4. Mica as an Insulation Material

The merits of mica as an insulation material in electrical engineering are a high dielectric strength as well as the stability of the dielectric strength at all electro technical occurring, relevant, temperatures. Only at the calcination temperature of 650 °C might the dielectric strength sustain losses. Mica is of great value when it comes to insulation materials for technical properties as well as price [10].

Electrical insulation contains a composite material which provides resistance against corona discharge due to the inorganic components (mica) and additionally improves mechanical strength with glass fabrics. Furthermore, thermally curable epoxy-based resins which serve as an organic binder are essential to laminate the glass fabrics and the mica compartments and concurrently prevent air inclusions within the insulation [22].

**Figure 1:**
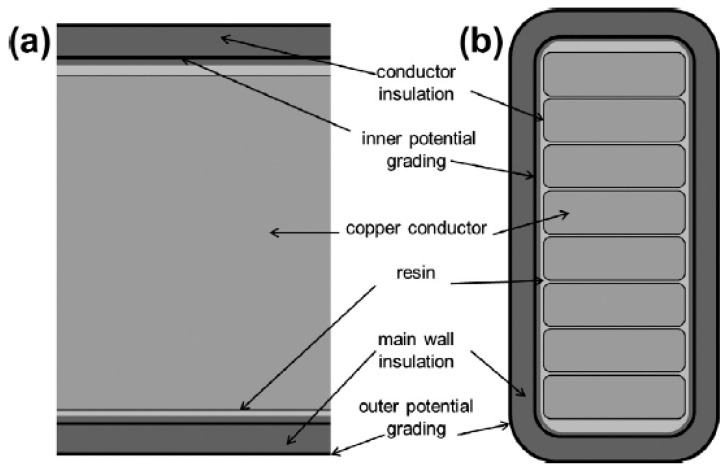
Construction of an electrical insulation. © copyright permission from Elsevier, 2014, Composites Part B: Engineering, New approaches towards the investigation on defects and failure mechanisms of insulating composites used in high voltage applications, License No. 3839281153636 [24].

The main wall insulation consists of about 65% mica paper, 25% resin and the glass fabric and other support materials compose 10% [23]. For the manufacturing of winding insulations of rotating machines, two technologies have been established. The vacuum pressure impregnation (VPI) process works in the following way: The mica tape, which is strengthened with a glass fabric, is wrapped around a copper conductor and hence forms the main insulation (roebel bar). Filed grading tapes are wound around the main insulation to improve corona resistance. The essential construction of such an electrical insulation is presented in Figure 1.

The roebel bar is semi-cured in an oven to remove any moisture or volatile components and afterwards moved to the vacuum pressure impregnation tank. Using vacuum, almost all the air from the insulation is expelled. An epoxy resin is added to the tank while vacuum is still used to fully cover the roebel bar. After releasing the vacuum, the material is pressurized to force the resin completely into the insulation material. After removing the bar from the VPI tank it is placed in an oven to cure the resin and fully develop the properties of the insulation system [25]. Another well-known technology is the RR (resin rich) technology. In this process, the mica tapes of the main wall insulation are consolidated by a resin which is solid at ambient temperature. These tapes, the so-called prepags, are manually wrapped around the mechanically formed copper conductors. During the heating step of the RR process, the required temperature as well as the pressure is utilized in a tank with an asphalt bath. This leads to a curing of the resin within the tape and as a further consequence to a solid composite material. Both technologies are suitable to achieve an inherent insulation quality with comparable properties providing that similar design and quality control are used [26,27].

The main difference between the two technologies is the setup and the manufacturing of the technical insulation system of the inductors. While the VPI system is only ready for use after impregnation and curing of the winding in an air circulated furnace, the “leg” of the resin rich inductor which is separately cured under temperature and pressure is already a functional and verifiable insulation system before the integration in the stator [28].

The advantage of the VPI inductor, respectively the VPI process, can be found in more cost-effective manufacturing in serial production. Furthermore, the hard inductor in the end winding is remarkably resistant against destruction by magnetic forces and the resulting movements causing subsequent failure of the insulation. Concerning the partial discharge resistance the extraordinary connection of the inductor to the iron core leads to a long-lasting operational life span. However, the investment for the VPI impregnation is quite high, therefore a high production number of the manufactured windings are necessary to achieve a final pay off. In contra-distinction, the manufacturing of resin-rich inductors is more sophisticated, which leads to an increase in costs for the single inductor. The resin-rich inductor offers a huge advantage though the validation of the inductors before integration in the stator implies higher guarantee during fabrication.

Both insulation systems are comparable concerning the product’s quality for the respective production process. Nevertheless, the constructive fringe conditions avoid exchange between the two technologies, which are also presented in Table 3 [29].

## 5. Insulation Failure in Power Generators

Due to the increasing demand of generating plants, rotating machines will definitely be an important element in the electrical energy supply. Therefore their impeccable functioning is of extreme importance for both producers of high voltage machines as well as for energy suppliers. Cost reduction caused by shorter maintenance times and higher operational life span is therefore of high interest [31].

In the last years, the evaluation of the magnitude of aging of large generator insulations has been of huge interest for several researchers [32,33]. Some findings were published in recent years containing the main conclusions of the insulation sate process and aging validation [34,35,36,37,38].

**Figure 2:**
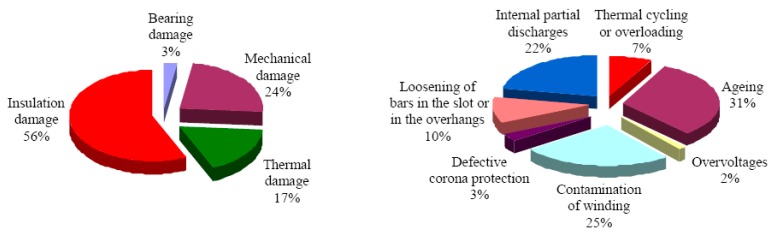
Percentage of the particular damages (**left**), damages divided into 7 distinct groups (**right**). © copyright permission from IEEE, 2008, IEEE Electrical Insulation Magazine, Insulation Failure Mechanisms of Power Generators, License No. 3844180528249 [46].

Several studies deal with causes of failure of high voltage rotating machines in general as well as the failure mechanisms of power generators [39,40,41,42,43,44]. The investigation of 1199 hydro generators was carried out by the CIGRE study committee SC11, EG 11.02 shows an example of 69 break down scenes [45]. In 56% the break downs were caused by a failure of the insulation. Other reasons were found in mechanical, thermal, or bearing damages (Figure 2 left). The main causes of the mentioned damages are divided into seven distinct groups (Figure 2 right).

Therefore it may be assumed, that the failure of the insulation is crucial in the breakdown of high voltage rotating machines [46].

The published findings of failures range from contamination of the winding insulation during fabrication to several aging processes [22,47]. Since the insulation is suspended by stress of various kinds like mechanical, electrical, ambient, and thermal stress during operation, the insulation may incur a loss of its dielectric and mechanical strength [33,48].

The insulation stress may be short term, respectively, accidental as well, as long term usage may lead to permanent damage and is associated with the operating regimes. Electrical stresses (normal, accidental) can cause partial discharges, electrical and water trees which might result in degradation and failure of the initial electrical characteristics of the insulation. Mechanical stresses (between two conductors or conductors and magnetic cores, *etc.*) induce abrasion and detachments of the insulation and might, as well, lead to cracks inside the material which ends in malfunction. Thermal stresses might determine weight loss, reduction of thickness, and insulation resistance to humidity and therefore change the required electrical and mechanical properties for the worse. The environmental stresses such as oxygen, humidity, radiation *etc.* might boost chemical reactivity and/or lead to new degradative reactions of the insulation [49].

The main difference between degradation and breakdown is the period of time. While degradation happens over a longer period, failure is a sudden event which is disastrous since the insulation is unable to support the nominal voltage after failure [50,51].

Fothergill published in 2006 an experimental study using samples of mica paper and epoxy resins which underwent simple and combined stresses with respect to the dependence of the capacity and the loss factor with regard to duration and strength of the stress situation [52].

**Figure 3:**
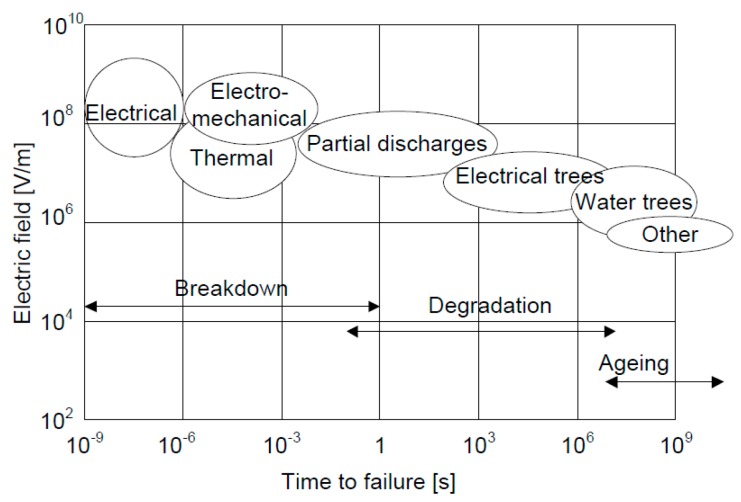
Values of electric field strength and the duration of certain mechanisms causing insulation damage. ^©^ copyright permission from the author(s), this is an open access article distributed under the terms of the Creative Commons Attribution License http://creativecommons.org/licenses/by/3.0/ [52].

Figure 3 represents the values of the electric field strength and duration of certain mechanisms which finally caused the insulation damage. Table 4 displays the process, respectively characteristic and the difference between breakdown, degradation and aging.

Another, more recent study was published in 2013, which shows all root causes for each of the 111 failure mechanisms which were identified at that time classified by their category of stress.

Table 5 shows failures which occurred due to both thermal stress and electrical stress. Ambient stress caused 35 failure mechanisms and 60 can be attributed to mechanical stress.

The number of intermediate physical states in these failure mechanisms is presented in Table 5 for each sort of stress category and the root causes per stress category are shown in Table 6 [53].

According to Fothergill [52], most of the researchers employed nondestructive methods like insulation resistance, polarization index, dielectric dissipation factor, and phase resolved distributions of partial discharge to evaluate impairment of insulations.

Since these measurements are only eligible for certain insulations or test conditions, the development of proper test methods has still been a topic of discussion among several researchers. The main topic was to find an appropriate testing system to evaluate the impairment condition of the epoxy/mica insulation and define parameters that give indication of the actual condition of the insulation [54,55,56].

Several studies from different groups were performed to investigate the failure mechanisms of the mica insulation. Sample bars or other test specimen were used in the published surveys [57,58]. A list, respectively a comparison of insulation testing methods is presented by Vogelsang [37].

The main result of several of the above mentioned surveys led to the assumption, that electrical breakdown indeed causes the final collapse of the electrical insulation, but apparently electrical stress is not the decisive factor when it comes to the aging of the insulation. In fact it is assumed, that thermal degradation of the binder resin controls the aging mechanisms, mechanical stress due to vibration, switching pulses and stress might be caused by decreased thermal expansion coefficients of the materials involved. The second main point of the studies is the temperature dependency. In the case when aging occurs under thermal, mechanical, and electrical stress at a moderate temperature of about 130 °C, it leads to an increase in the lifetime, whereas a temperature up to 180°C results in a rapid decrease in the lifetime.

The results are indicative of an increasing thermal degradation of organic matter on the one hand and a decrease of internal stress and crack formation of the binder resin at higher temperatures on the other hand [46].

## 6. Epoxy Resins as Binder for Insulation Composites

The most common resin systems are epoxy resins due to their excellent adhesion, permeability, corrosion resistance, and mechanical properties. Additionally, they distinguish themselves by an outstanding combination of physical and electrical properties compared to polyurethane-, silicone-, alkyd, unsaturated polyester-, and phenol-resins [59].

The majority of epoxy resins are used through polyaddition as a cold-hardening (room temperature) system, respectively as a hot-hardening system up to 200 °C. Besides the mainly used polyaddition reaction, several different polymerization mechanisms (cationic or coordinative mechanisms) are known for the cross linkage of epoxy resins [59,60].

**Figure 4:**
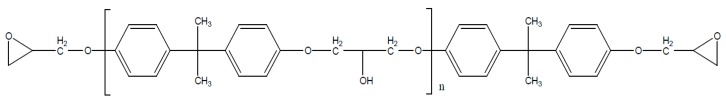
Diglycidylether of Bisphenol A (DGEBA) *n* = 0–1.

**Figure 5:**
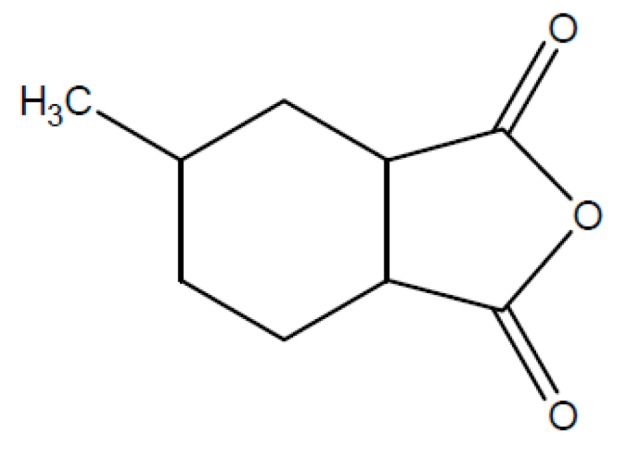
4-Methylhexahydrophthalacid anhydride (MHHPA).

For the winding of electrical machines (e.g., motors, generators) the diglycidylether of Bisphenol-A (DGEBA, Figure 4) in combination with different hardeners- and accelerator components is mainly deployed. For VPI resins anhydride hardening, e.g. 4-methylhexahydrophtalacid anhydride, (MHHPA, Figure 5) is preferred. Among other things, the properties can specifically be changed by the molar mass of the resin component as well as the processing conditions. The mostly used electro technical resin system DGEBA/MHHPA is easily applicable for use in the vacuum pressure impregnation process (VPI) by addition of different accelerators [12,61,62].

**Figure 6:**
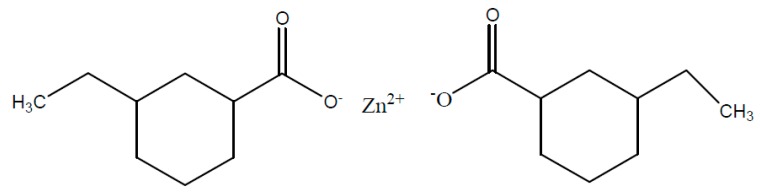
Zinc naphthenate (ZnNaph); structure example.

Referring to the literature, numerous distinct metal salts accelerators can be used for epoxy/anhydride systems, which can lead to a huge difference concerning the hardening temperatures (90–170 °C) as well as storage stabilities [63,64]. The usage of different accelerators can give rise to high levels of cross-linkage which leads to a shift of the glass transition temperature. Within the insulation composite of rotating electrical machines, zinc naphthenate (Figure 6) is mainly used as accelerator for the DGEBA/MHHPA system [65,66].

**Figure 7:**
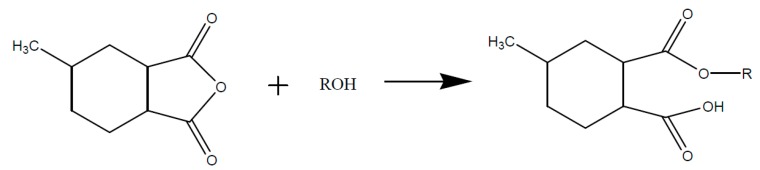
Opening of the anhydride ring due to reaction with an alcohol (R-OH).

A cyclic anhydride like e.g., MHHPA however does not react directly with the epoxy group. The anhydride ring has to be opened by an active hydrogen atom, a hydroxyl group, or a Lewis base, as shown in Figure 7.

**Figure 8:**
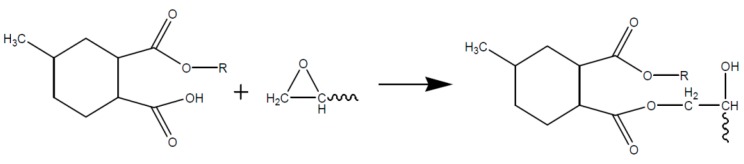
Reaction of the opened anhydride with an epoxy upon formation of an ester and a hydroxyl group.

The resulting organic acid is then able to react with the epoxy group to form an ester, as shown in Figure 8.

**Figure 9:**
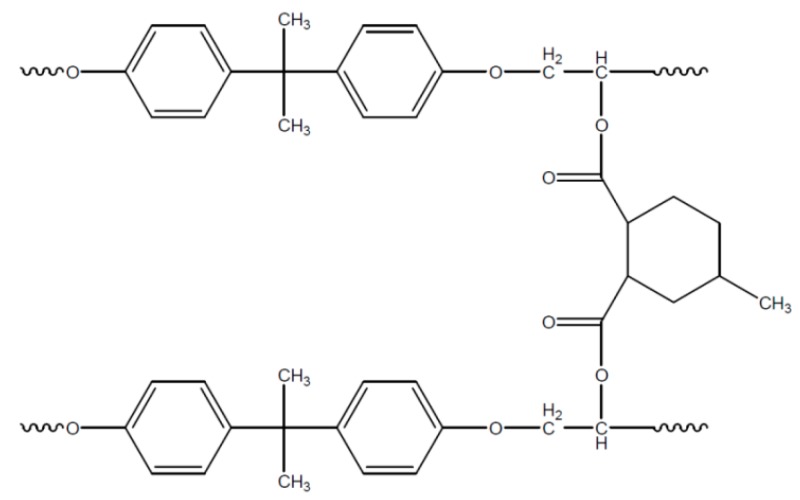
Network structure of an epoxy resin based on DGEBA/MHHPA.

Due to a further reaction step of the formed hydroxyl group with an anhydride the step reaction is continued. If di-epoxy-monomers (DGEBA) are used, a three dimensional network structure is formed, as represented in Figure 9 [67]. The molecular ratio between epoxy groups and anhydride groups therefore should be 1:1 to avoid rest monomers and ensure that all hydroxyl groups take part in the reaction.

**Figure 10:**
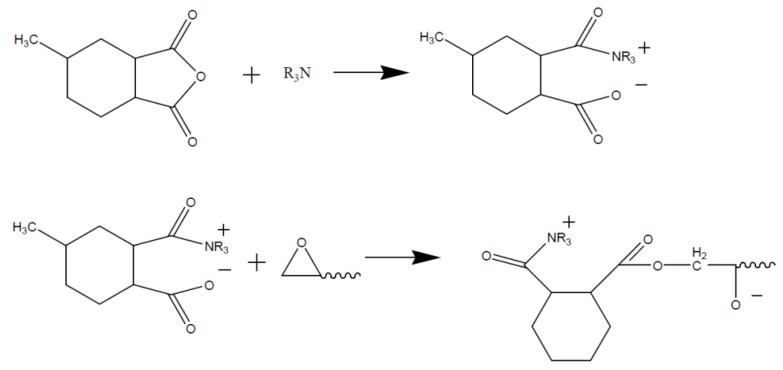
Amine-accelerated reaction of anhydride with an epoxy.

For the anhydride-hardening, tertiary amines are mainly used as reaction accelerators. According to the literature, those amines do not function as typical catalysts since they do not revert back to their original structure (Figure 10) [68].

**Figure 11:**
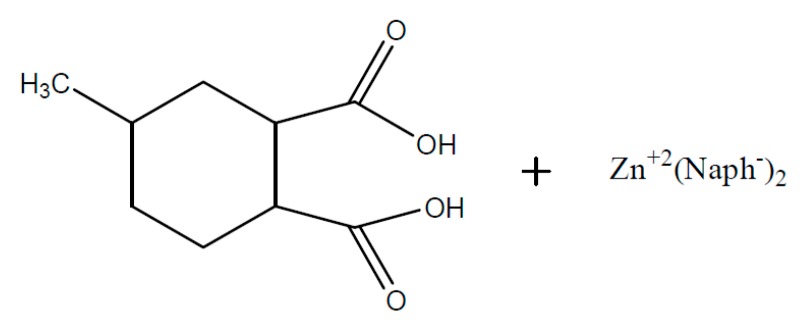
Opened anhydride and zinc naphthenate (Naph^−^ = R–COO^−^).

Another, essentially more complicated possibility represents the reaction of anhydrides with epoxy in the presence of metal salts (e.g., zinc naphthenate) as an accelerator. Since the epoxy/anhydride-system contains water in small amounts and furthermore aliphatic hydroxyl groups are found in DGEBA-structures at *n* =1, an opening of the anhydride ring to a carbon acid can be expected to a slight extent. Therefore the initiation of the hardening reaction of the presented carboxylic acid in Figure 11 in the presence of zinc-carboxylate as a catalyst can take place.

**Figure 12:**
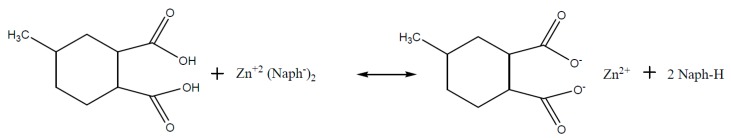
Zinc-naphthenate–MHHPA—dissociation at higher temperatures.

In respect of the reaction mechanism, Blank [69] and Han [70] assume that the reaction equilibrium between carboxylic acids and zinc naphthenate (Figure 12) at low temperatures is located on the left side. The reactivity is low and the epoxy/anhydride/ZnNaph system represents only a low increase of viscosity at room temperature.

**Figure 13:**
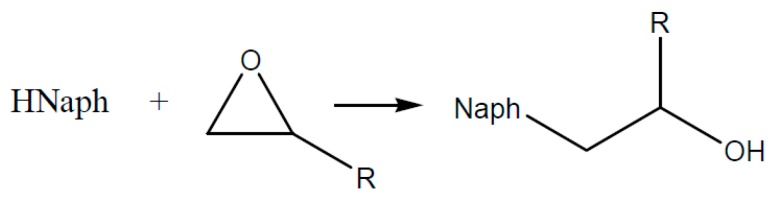
Epoxy-ring opening via free naphetenic acid H–Naph.

If the temperature is increased, a faster exchange presented in Figure 12 of the reaction partners will occur and the equilibrium shifts to the right side. (Formation of free naphthenic acid, H–Naph). Due to the reaction of the free H–Naph with an epoxy group (Figure 13) the reaction equation of Figure 13 shifts further to the right side and leads to the formation of more (R’COO)_2_Zn [69,70].

The formed naphthenic acid is able to react with an epoxy (Figure 13).

**Figure 14:**
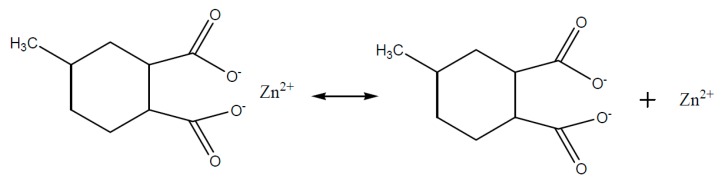
Equilibrium: non-dissociated and dissociated zinc salt of MHHPA.

**Figure 15:**
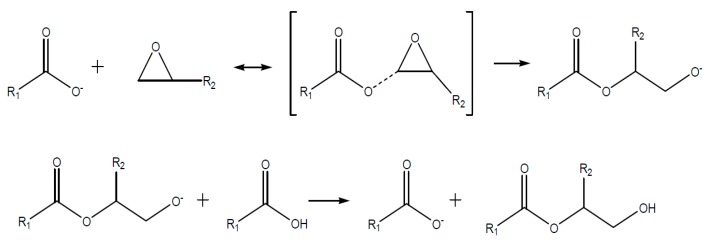
Carboxylate–epoxy reaction.

In Figure 14 the equilibrium between none-dissociated and dissociated zinc carboxylate (R’COO)_2_Zn is represented. The free carboxylate, which is formed of MHHPA, then reacts as a nucleophile and ring opening with the epoxy group and an ester group is formed (Figure 15) [69].

In the middle 1960s, Siemens AG developed the epoxy/anhydride system in combination with mica tapes and the therein immobilized zinc naphthenate as an accelerator for the VPI technology. This system is still used today [71,72]. Cycloaliphatic anhydrides like MHHPA react—on the contrary to aminic hardeners—at higher temperatures and reveal good storage stability. The combination of DGEBA with MHHPA exhibits a very slow reaction with the result that an accelerator is required [65,70].

## 7. Alternative Resins as Binder Materials (Cyanate Resins)

Cyanate resin systems represent another option for high voltage insulation. Compared to epoxy systems, cyanate resins exhibit a higher viscosity as well as higher glass transition temperatures. The advantages are excellent insulation properties and a very high temperature resistance [73,74,75].

**Figure 16:**
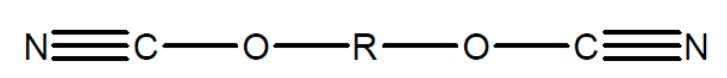
Structure of dicyanate ester.

Typical starting products for cyanate resins are bisphenol A, tetra methyl bisphenol F, bisphenol M or phenolic novolac. The reaction with cyan acids or gaseous cyan chloride leads to the reactive dicyanate ester Figure 16.

**Figure 17:**
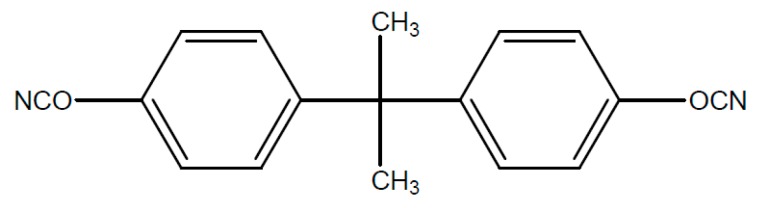
Structure of cyanate ester of bisphenol.

**Figure 18:**
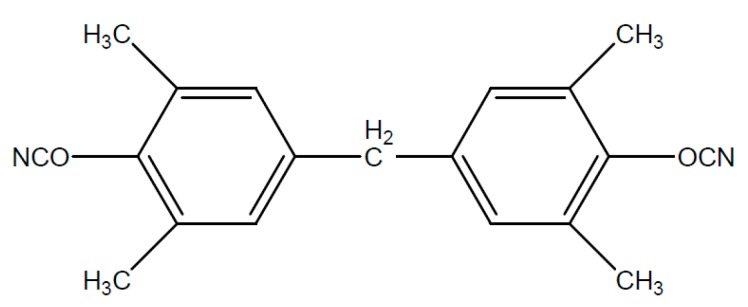
Structure of cyanate ester of tetra methyl bisphenol F.

**Figure 19:**
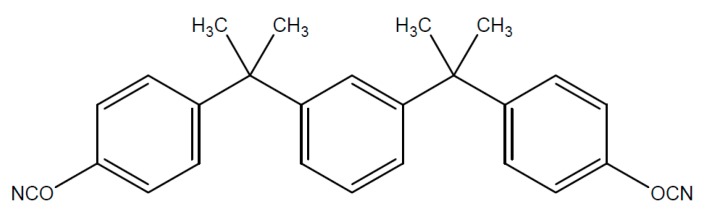
Structure of cyanate ester of bisphenol M.

**Figure 20:**
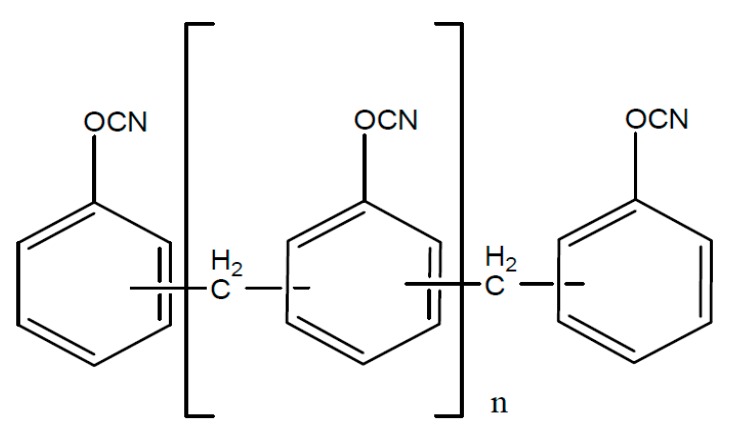
Structure of cyanate ester of phenol novolac resin.

The dicyanate esters, which are formed from the previously mentioned starting products are represented in Figure 17, Figure 18, Figure 19 and Figure 20.

**Figure 21:**
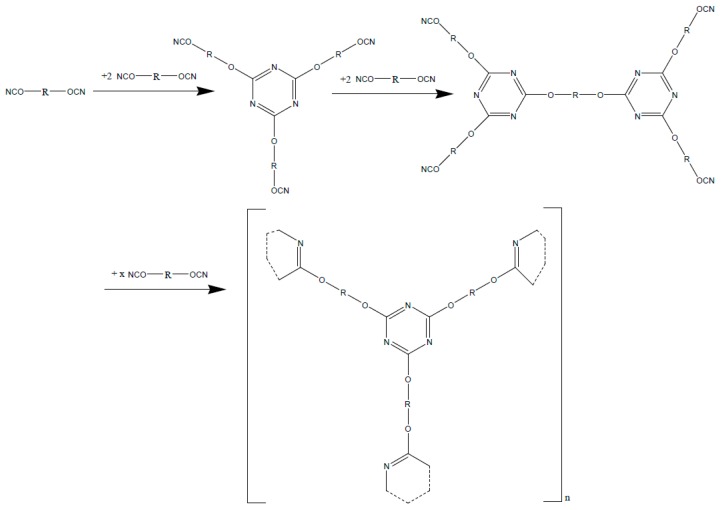
Polycyclic trimerization of di-cyanate ester to triazine-structures.

The hardening is achieved by a polycyclic trimerization of the dicyanate ester among the formation of triazin-ring (cyan urate) to a 3 dimensional network (Figure 21).

## 8. Temperature Resistance of Epoxy Resins

The different structures of the hardened epoxy resins lead to huge differences in the temperature resistance. Beside the different monomers, the chemistry of hardening and the resulting structure of the polymer main chain are essential for the temperature resistance [76].

**Figure 22:**
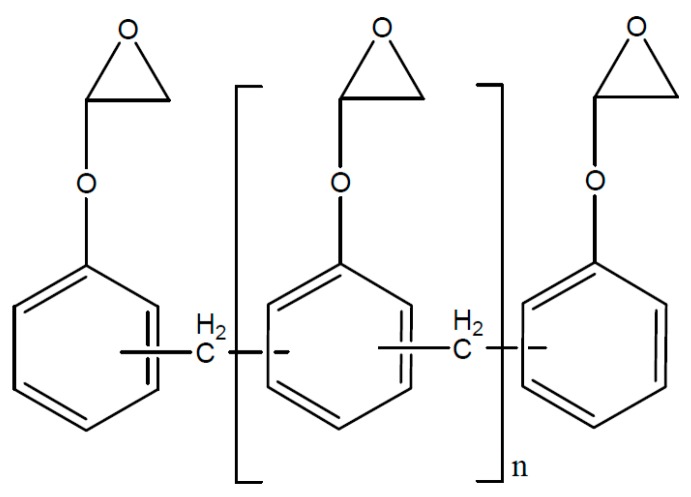
Epoxidized novolac.

Basically, polymers with high aromatic content display a better thermal resistance compared to polymers with high aliphatic content in the main chain. The reason is the high stability of aromatic systems. The inertia concerning chemical reactions can be explained by the resonance stabilization of aromatic carbon hydrogens. Therefore, epoxy resin systems with epoxidized novolac (Figure 22) display higher, thermal resistance compared to epoxy resins systems based on diglycidylether or bisphenol A.

**Figure 23:**
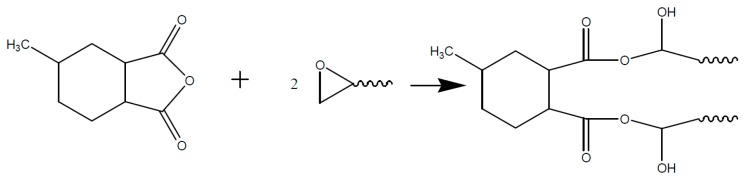
Hardening of epoxy/anhydride (simplified).

**Figure 24:**
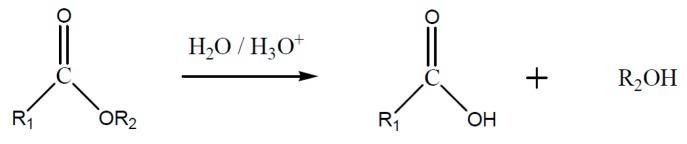
Hydrolysis of the ester structure.

**Figure 25:**
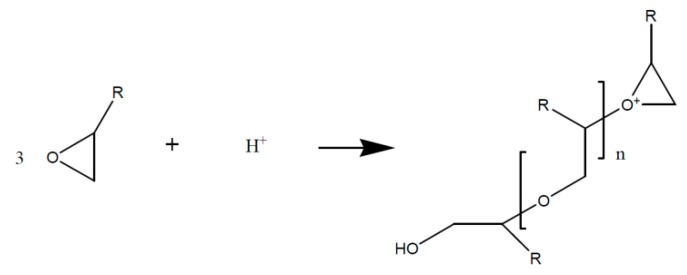
Polyether structure of the cationic hardened epoxy resins.

Besides the deployed monomer, the hardening reaction determines the main chain structure and as a further consequence the thermal resistance. The polyester main chain, which originates from an epoxy/anhydride-hardening, (Figure 22) tends to hydrolytic cleavage and results in chain break. The hydrolysis of an ester group is represented in (Figure 23). In the case where the polyether main chain is achieved by homo-polymerization of the epoxy resin, represented in Figure 24, the thermal resistance is increased because the polyether main chain (Figure 25)—in comparison to ester—cannot be cleaved by hydrolysis. In a similar way, the amine-hardened epoxy systems are stable against hydrolysis [77].

Concerning temperature resistance of epoxy resins the disruptive breakdown within the insulations composite occurs as a consequence of physical and chemical impairment. Besides thermal stress, strong potential occurs in the composite material due to different expansion coefficients of [57,77,78].

Thermosetting resins, particularly epoxy resins, have always played a significant role in certain applications such as the covering of surfaces, electronic components, and power moldings and they have served as matrix resins for advanced composites ever since. The adaptability of the formulation is another advantage of epoxy resins which allows their use as insulating materials [79,80].

The main insulation of high voltage electric machines is based on the VPI process. As previously mentioned, the conductors are swathed in a mica tape which contains an accelerator, zinc naphthenate, a resin, and the hardener. The tape itself is composed of a thin, non-calcinated muscovite tape, a glass fabric, a strengthened material, which links the glass fabric to the tape, as well as an accelerator [81]. 

This composite distinguished by a high specific stiffness and strength and in addition a high thermal stability. The molecular structure of the matrix is crucial for the required mechanical properties when used in high temperature ranges or at high levels of compressive stress at ambient temperatures, and therefore contributes considerably to the performance of the composite material itself [82].

A study published in 2011 shows an extensive characterization of the mica/epoxy-composite, its components as well as the thermo-analytical interactions between them. The thermal analysis was performed using the pure components and the results were consistent with those found in the literature [83].

A characterization of the pure components and the mixtures present in the composite has been performed using TG to gain information about thermal characteristics of each raw material.

**Figure 26:**
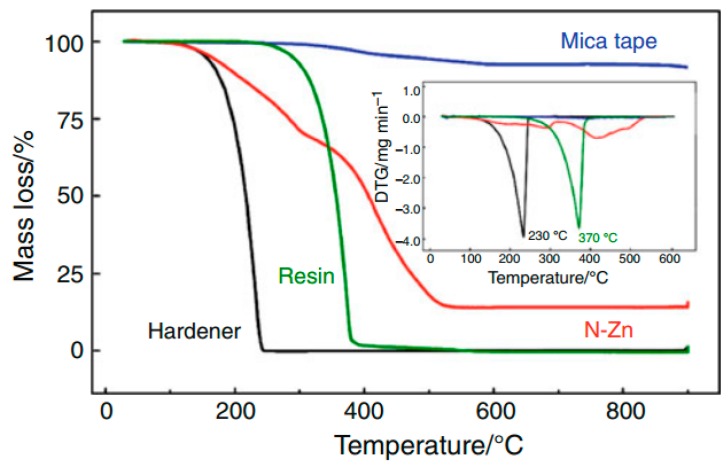
TG/DTG curves of components obtained under N_2_ atmosphere (10 °C·min^−1^), PT pan, and β of 10 °C·min^−1^. ^©^ copyright permission Springer, 2011, Journal of Thermal Analysis and Calorimetry, Thermal characterization of mica–epoxy composite used as insulation material for high voltage, License No. 3837180507265 [83].

Figure 26 displays the TG/DTG curves of the components. The results were compared to those found in the literature, which is presented in Table 7.

**Figure 27:**
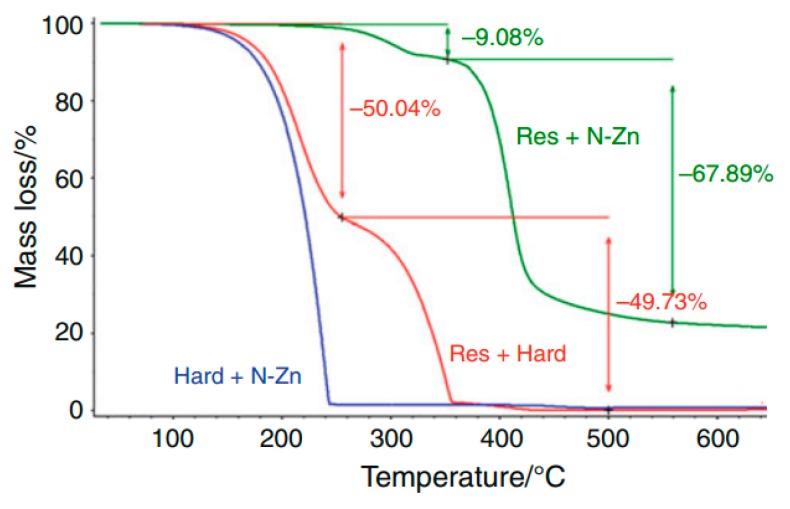
TG curves of mixtures: resin/hardener, hardener/N–Zn, and resin/N–Zn under N_2_ atmosphere (10 mL·min^−1^), Al_2_O_3_ pan, and β of 10 °C mL·min^−1^. ^©^ copyright permission Springer, 2011, Journal of Thermal Analysis and Calorimetry, Thermal characterization of mica–epoxy composite used as insulation material for high voltage, License No. 3837180507265 [83].

**Figure 28:**
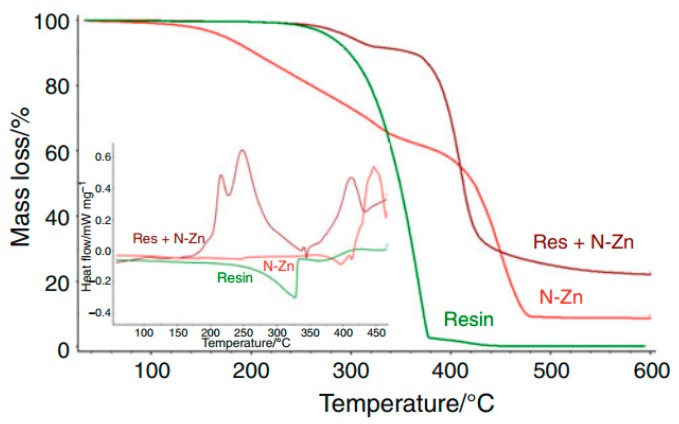
TG curves of resin, N–Zn and resin/N–Zn under N_2_ atmosphere (10 mL·min^−1^), Al_2_O_3_ pan, and β of 10 °C mL·min^−1^ and DSC curves under N_2_ atmosphere (50 mL mL·min^−1^), Al pan, and β of 10 °C min^−1^. ^©^ copyright permission Springer, 2011, Journal of Thermal Analysis and Calorimetry, Thermal characterization of mica–epoxy composite used as insulation material for high voltage, License No. 3837180507265 [83].

Comparing the TG/DTG curves of the components (Figure 26), the mixture of hardener with resin (1:1) (Figure 27) does not exhibit any detectable interference of the accelerator in the heating process relating to loss in mass. The pure hardener shows a mass loss at almost the same temperature. The mixture of resin/hardener (1:1) shows the same degradation pattern of both pure components, representing two events at onset 200 °C from the hardener and 320 °C, approximately from the resin. Moreover, it can be observed, that in the mixture of resin with zinc naphthenate (1:0.03) there is a huge mass loss at 360 °C, which can be attributed to a thermal decomposition of the polymer. The polymerization takes place between 180 and 280°C as shown in the resin/N–Zn mixture DSC curve in Figure 28 represented by two exothermic events (*T* = 210 and 260 °C) in a temperature range where the TG-curve does not show any mass loss. Furthermore, the DSC curves (Figure 28) of the pure components do not show any exothermic reaction until 300 °C. 

Therefore it can be assumed, that the accelerator is able to open the epoxy rings of the DGEBA and initiate a polymerization reaction with a small amount of N–Zn. The DSC curve of the mixture of the resin with the zinc naphthenate (resin/N–Zn) shows that at the end of the polymerization, the partial resin evaporation is evidenced by an endothermic event occurring at a temperature of 344 °C.

**Figure 29:**
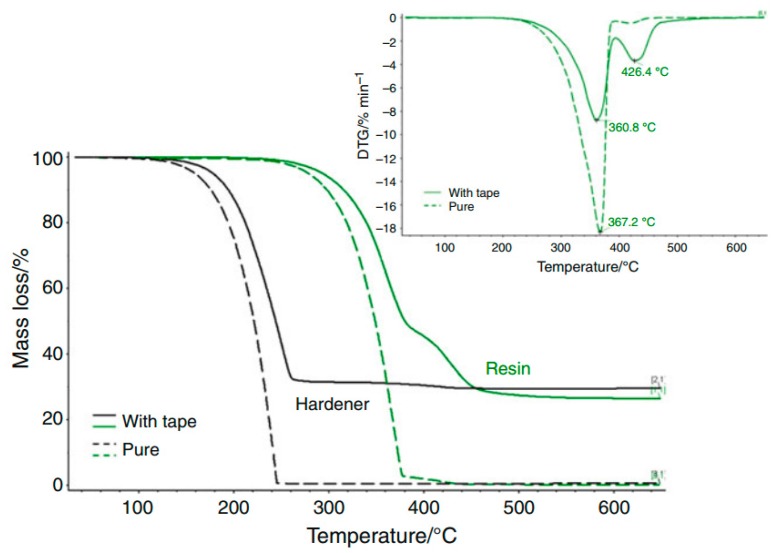
TG/DTG curves of resin and hardener with and without mice tape under N_2_ atmosphere (10 mL·min^−1^), Al_2_O_3_, pan, and β of 10 °C·min^−1^ .^©^ copyright permission Springer, 2011, Journal of Thermal Analysis and Calorimetry, Thermal characterization of mica–epoxy composite used as insulation material for high voltage, License No. 3837180507265 [83].

Figure 29 shows the TG and DTG curves of the resin and the hardener with and without the mica tape. As can be seen from Figure 27, the hardener does not show any interaction with the tape, representing the same shape and profile in pure form as also with the tape. Referring to the physical obstacle of evaporation and decomposition, the substance with the tape shows a slight shift of the degradation temperature. Moreover, the interaction of resin and hardener with the tape shows an exothermic reaction at 426 °C which can be seen as well using DTG. This leads to the assumption, that between these two components, the epoxy ring might open up and the molecules can react. The polymerization then shows a decomposition event at this higher temperature.

All in all, the published thermal analysis allows a detailed study of curing processes and thermal decomposition and therefore allows the prediction and suggestion of mechanisms as well as further optimization of the system. The glass transition (*T*_g_) of the composite showed a value of *T*_g_ = 138 ± 2 °C which finally characterizes the mica–epoxy composite material [83].

## 9. Final Review

Mica is widely spread in nature and has been known for many years, but gained increasing importance due to the developments in the electrical industry. Due to its unique properties in many respects, mica represents the optimal material for many applications. Especially for the purpose of electrical insulation, mica is superior to all comparable materials because of its extraordinary performance and is therefore widely deployed in high voltage rotating machines.

A typical, electrical insulation contains a composite material which consists of a mica tape and a glass fabric. An epoxy-based resin serves as an organic binder and laminates the two components. The main wall insulations in high voltage applications consists of about 65% mica, 25% resin, and 10% other support materials.

Due to the increasing demand of power plants, rotating machines definitely demonstrate an important element when it comes to electrical energy supply. Therefore, impeccable functioning is of immense importance. In the last years, the evaluation of the magnitude of aging of large generator insulations has been of huge interest for several researchers. Various test methods have been developed to gain more information about insulation failure mechanisms.

Due to these intense studies and comparison of mica-epoxy-composites, the properties of the pure components as well as the composite and its failure mechanisms, a better understanding and therefore a prospective optimization of high voltage insulations might be enabled.

## Figures and Tables

**Table 1:** Summary of properties of muscovite and phlogopite [6].

Properties	Unit	Muscovite	Phlogopite
**Thermal properties**			
Melting temperature	°C	1,200 to 1300	1,200 to 1,300
Start of Calcination	°C	550 to 650	750 to 900
Long term thermal stability	°C	500	700
Thermal conductivity	W/m·K	0.25 to 0.75	*ca.* 1.7
Coefficient of thermal expansion	K^−1^	90 × 10^−7^	135 × 10^−7^
Specific heat	J/g·K	0.06	0.26
Flammability		inflammable	inflammable
**Mechanical properties**			
Density	g/cm^3^	2.6 to 3.1	2.6 to 3.2
Hardness (acc. to mobs)	N/mm^2^	2.8 to 3.2	2.5 to 2.7
Compressive strength	N/mm^2^	200 to 400	150 to 300
Shear strength	N/mm^2^	250	110
E-module (*d* = 250 µm)	N/mm^2^	180 × 10^−3^	170 × 10^−3^
**Electrical properties**			
Permittivity	ε	6 to 8	5 to 6
Dielectric loss factor (tan α)		3 × 10^−4^ (10^6^ Hz)	10 to 100 × 10^−4^
Dielectric strength (20 °C, 50 Hz)	KV/mm	60 to 70 (up to 1 mm)	50 (up to 1 mm)
Resistance to tracking	KB-wert	>600	>600
Corona resistance		corona resistant	corona resistant
**Other physical/chemical properties**	****		
Refractive number		1.56 to 1.61	1.58 to 1.61
Radiation resistance		very good	very good
Resistance against organic solvents		resistant	resistant
Acid resistance		resistant (except hydrofluoric acid)	resistant (except hot acids)
Oil resistance		resistant	resistant
Color		reddish ,green, colorless, brown	amber, green
Physiological effect		no precautionary measures, harmless	
Amount of crystal bonded water %		4.5	3

**Table 2:** Application of mica [6].

Application/Devices	Example of usage
electrical devices	inductor of voltmeters, commutators, power inverters, high voltage commutators, rotating field coils, high voltage transformers, heat traps
radio receiver, TV, radar	solid state systems, condensers, tubes, microwave windows, transistor shielding
electrical light devices	arc lamps, huge incandescent lamps, bases for lampshades, neon lamps, dimmer counters, turn signal systems
mixed electric applications	fuse cover platelets, spark plugs for high compression engines, sealing shims, insulators
electrical household appliances	coffee machines, cigar lighters, hair roller, irons, immersion heaters, permanent wave devices, toasters, vibrators, space heaters, hair dryer, waffle iron
electrical monitoring systems	grid resistors, pyrometer, relays, electrical and thermal controller
mechanical applications	dials, membranes for acoustic instruments, heart-lung-machines, respirators, gaskets for high temperature measurement instruments, lantern windows, fireplaces, unbreakable safety goggles, quarter-wave-plates for optical instruments, vision panels in ovens, synthetic, optical crystals
industrial electrical applications	corrugated rolls, glue pots, lead baths, devices for local warming, several heating elements, soldering irons, thermostats

**Table 3:** Comparison of vacuum pressure impregnation (VPI) and resin rich [30].

	VPI	Resin rich	
		With heated molds	With asphalt pressure molding
**Strand insulation—dielectric strength**	negligible difference between systems		
**Insulation tape**	mica paper with glass fabric carrier and without resin	mica paper with resin and glass carrier	mica paper with resin and glass carrier
**Number of insulation layers**	depending on rated voltage no appreciable difference between systems		
**Internal potential grading for optimized field distribution in the main insulation**	yes	multiturn coils—no Stator bars—yes	yes
**Corona protection with tapes**	yes	yes	yes
**Vacuum impregnation with epoxy resin**	yes	yes	yes—resin pre-loaded in tapes
**Winding overhang section of bar**	winding overhang and slot section—same materials	winding overhang and slot section—different material for coils only to improve windability	winding overhang and slot section—same materials
**Composition of main insulation** **Mica content** **Glass content** **Resin content**	approx. 65% approx. 10% approx. 25%	approx. 65% approx. 10% approx. 25%	
**Main insulation dielectric strength**	negligible difference between systems		
**Partial Discharges within the insulation (PD level)**	very low, no micro voids; PD/single bar lower than 2 nC	pressed—slightly higher due to lack of vacuum	very low, no micro voids; PD/single bar lower than 2 nC
**Advantages**	void-free insulation by removal of air through the vacuum process-penetration of impregnating resin into the insulation to fill the voids, minimization of corona activity, achieve very low levels of PD, high temperature capability	low viscosity resin during heating results in very few retained voids, insulation system results in very high dielectric strength, ow PD system, high temperature capability	void-free insulation by removal of air through the vacuum process, presence of pre-impregnated tapes ensures maximum void fill particularly near bare bar, achieve extremely low levels of PD, high temperature capability

**Table 4:** Process/characteristic and the difference between breakdown, degradation and aging [53].

Process/Characteristic	Breakdown	Degradation	Aging
**Evidence**	direct observation (normally by eye-hole through insulation)	observable directly (may require microscopic or chemical techniques)	difficult to observe (may even be difficult to prove existence)
**Place**	continuous filament	occurs in weak parts	assumed to occur throughout insulation
**Size**	< mm (dependent on energy of event)	<µm (may form larger structures)	<nm (molecular scale)
**Speed**	fast (occurs in <<1 s)	less than required service life (hours—years)	continuous process (whole service life)
**Effect**	catastrophic (insulation cannot be used afterwards)	leads to breakdown (reduces breakdown voltage)	may lead to degradation (may not reduce breakdown voltage)
**Examples**	thermal, electromechanical, mixed mode, avalanche, intrinsic	partial discharges, electrical trees, electrochemical trees	bond scissions, nano voids, trap formation, non-electrical changes (oxidation *etc.*)

**Table 5:** Process types and the distribution of the physical states [53].

Types of process	Number of physical states
Thermal (t)	9
Electrical (e)	22
Ambient (a)	14
Mechanical (m)	35

**Table 6:** Root causes per stress category and the distribution of failure mechanisms [53].

Root causes per stress category	Number of failure mechanisms
**Thermal Stress (T)**	8
T1 thermal aging (normal operation)	3
T2 accelerated aging (operation above specified rated temperatures)	3
T3 aging due to thermal cycling (frequent start/stop operation)	2
**Electrical Stress (E)**	8
E1 improper manufacturing or design of bars	2
E2 poor semiconducting coating on the straight part of the bars (slot discharges)	1
E3 poor design or manufacturing of end winding stress grading material (corona discharges)	1
E4 insufficient spacing between end windings (gap discharges)	1
E5 overvoltage transients	3
**Ambient Stress (A)**	35
A1 conducting contamination (carbon, steel or copper dust)	6
A2 non-conductive contamination (construction dust or oil)	9
A3 moisture in ambient air	7
A4 abrasive material attack	3
A5 water leakage (cooling system failure, fire protection and spills)	10
**Mechanical Stress (M)**	60
M1 loose windings	17
M2 bad connection	6
M3 presence of external objects or loose parts	5
M4 mechanical shocks	4
M5 Projectiles	4
M6 Rotor and/or stator deformation	24

**Table 7:** Data of the single materials (experimental *vs.* literature) [83].

Component	Degradation temperature (°C)	
	Experimental	Literature
Resin	322	>300
Hardener	207	203
Mica tape	348	–
N–Zn	251	250
